# Mesenteric Shearing in a Pediatric Patient, Successfully Managed Conservatively: A Case Report

**DOI:** 10.1055/a-2642-0650

**Published:** 2025-07-15

**Authors:** Karim Sabeh-Ayoun, Nour Bakhos, Mustafa Natout, Ahmad Zaghal

**Affiliations:** 1 Department of Surgery, American University of Beirut Medical Center, Beirut, Lebanon; 2 Department of Diagnostic Radiology, American University of Beirut Medical Center, Beirut, Beirut Governorate, Lebanon; 3 Division of Pediatric Surgery, Department of Surgery, American University of Beirut Medical Center, Beirut, Lebanon

**Keywords:** mesenteric shearing, mesenteric hematoma, pediatric trauma, case report

## Abstract

Mesenteric shear, injury due to sudden stretch of the mesentery, is a rare condition that remains poorly studied, especially in the pediatric population. Often resulting from trauma, its presentation can vary from nonspecific and vague abdominal symptoms to an acute abdomen and peritonitis requiring urgent surgical intervention. Unified management strategies are not yet in place. We describe the case of a 10-year-old boy who presented to the emergency department with diffuse abdominal pain of 1 day duration, 2 days after he sustained blunt abdominal trauma with an elbow during a soccer match. He was hemodynamically and clinically stable. Computed tomography scan revealed soft tissue thickening in the left upper quadrant at the root of the mesentery with mild surrounding inflammatory mesenteric fat stranding, suggesting mesenteric shearing. With stable vitals, a soft abdomen on physical exam, and no drop in hemoglobin, the decision was made to treat the patient conservatively. The patient was admitted for observation, and after frequent abdominal exams, stable laboratory results, and abdominal imaging, he was discharged home without any surgical intervention. Conservative management can be successful in the case of a stable patient without alarming physical, laboratory, or imaging findings. Observation and close monitoring remain essential to detect complicated cases that require surgical intervention.

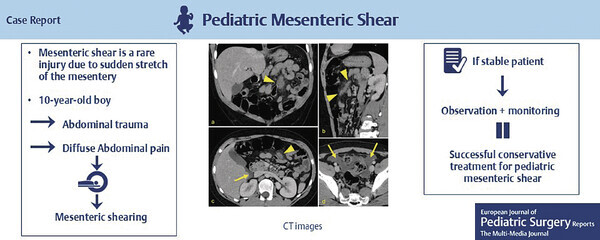

## Introduction

Mesenteric shear forces can result in mesenteric injury manifesting as mesenteric hematomas. Very few studies touch on this type of injury, especially after blunt abdominal trauma in the pediatric population. Therefore, it comes as no surprise that consensus on the management of mesenteric shearing and hematomas in pediatrics is not yet at hand. For many, just identifying the injury is enough to warrant operative measures for further evaluation or management. 1 However, as of more recently, the safety and appropriateness of deferring surgery in benign abdominal trauma patients with nonworrisome clinical findings is being stressed. 2

## Case Presentation

A 10-year-old boy is brought into the emergency department for complaints of diffuse abdominal pain. The persistent pain began 1 day prior to presentation, was moderate to severe, and worsened with abdominal flexion, coughing, and sneezing. Medical and surgical history was negative for any conditions or procedures, except for some form of mild atopic airway disease. Further questioning revealed a history of abdominal trauma with an elbow to the abdomen during a soccer match 1 day before the pain began (2 days prior to presentation).

On physical exam, the patient was awake, alert, and comfortable. His abdomen was flat and soft, with mild-to-moderate focal points of tenderness and rebound tenderness on palpation of each abdominal quadrant. Other than mild tachycardia in the absence of pain control, vital signs were all within normal. Laboratory work-up revealed an elevated C-reactive protein (50.9 mg/L; 0.0–2.5 mg/L), increased neutrophil and decreased lymphocyte proportion (73 and 13%, respectively; 40–65 and 25–40%, respectively) and a mildly decreased serum carbon dioxide level (21 mmol/L; 24–30 mmol/L). The hemoglobin (Hg) level was just below the lower limit of normal (11.7 g/dL; 12.0–15.0 g/dL), and the white blood cell count was at the upper limit of normal (11,900 cu mm; 4,500–13,500 cu mm).

**Fig. 1:**
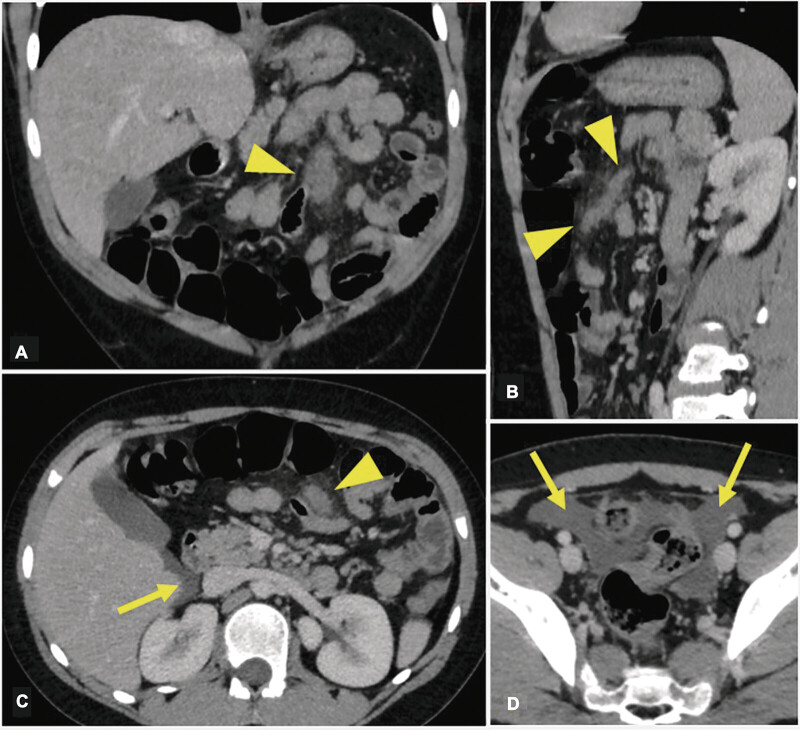
Contrast enhanced CT scan of the abdomen performed on December 25, 2022, shows an ill-defined oblong dense mesenteric root structure (arrowhead) in the left para-sagittal aspect of the upper abdomen, on the coronal ( **A** ), sagittal ( **B** ), and axial ( **C** ) reformats, representing mesenteric shearing. There is associated small amount of dense intraperitoneal fluid in the perihepatic space (arrow in C) and in the pelvis (arrow in **D** ), representing blood.

The decision was made to perform intravenous (IV) contrast-enhanced venous phase computed tomography (CT) imaging of the abdomen and pelvis. The images showed a focal area of oblong soft tissue thickening in the left upper quadrant at the root of the mesentery measuring 4.7 cm × 2.3 cm × 0.8 cm, with mild surrounding inflammatory mesenteric fat stranding, suggestive of blood products or mesenteric shearing ( Fig. 1 ). There was associated trace dense free fluid in the hepatorenal space, along the paracolic gutters, in the lower quadrants, and in the pelvis, suggestive of blood. Multiple mildly enlarged mesenteric lymph nodes were also present along the root of the mesentery and in the right lower quadrant, probably reactive.

It had been around 24 hours since the pain began, and the patient was hemodynamically and clinically stable. Imaging did not show signs of active bleeding, and a repeat CBC showed unchanged Hg levels. Conservative management was thus considered to be a sufficient and safe approach, and the decision to proceed as such was taken after thorough discussion with the patient's parents. The patient was admitted for observation and monitoring, with frequent abdominal exams, vital checks, Hg trending, and a repeat CT with IV contrast approximately 24 hours after the initial CT. The pain decreased, and the abdominal exam improved. Vitals remained within normal, Hg trends were stable, and the repeat CT showed no significant changes. After 44 hours, the patient was deemed stable and was discharged with instructions to repeat CBC and follow-up. Around 10 days after discharge, the patient developed high-grade fever in association with chills, headaches, sore throat, worsening cough, and worsening abdominal pain. Abdominal exam showed a soft abdomen with mild suprapubic and right lower quadrant tenderness. A CT with IV contrast of the abdomen and pelvis re-demonstrated the oblong soft tissue structure in the mesentery, this time seen in the mid abdomen below the umbilicus and mildly extending to the right lower quadrant. Minimal decrease in size, decrease in central density (possibly related to blood liquefaction), and some residual surrounding inflammatory changes were noted. The lower chest showed findings suggestive of a pulmonary infection, and management was as an outpatient accordingly. Follow-up of the patient 2 years after the incident revealed no signs or symptoms concerning for the development of complications.

## Discussion

Studies showing the incidence of mesenteric hematomas are lacking. The proportion of mesenteric injury in patients 16 years of age or less presenting acutely with blunt abdominal trauma is around 1%. 3 Thus, it is not surprising that consensus on the management of mesenteric hematomas in the pediatric population is far from reach.

Hollow viscus injuries are associated with significant morbidity and mortality and are almost always managed surgically. 4 These injuries can be associated with mesenteric abnormalities on CT after blunt abdominal trauma. 5 This appears to suggest that the finding of mesenteric hematomas warrants an operative approach to management. However, it may be that for hemodynamically stable patients without signs of peritonitis, a nonoperative initial approach is safe, as might be suggested by a retrospective cohort study assessing the presence of isolated free intraperitoneal fluid in pediatric victims of blunt abdominal trauma. 2 In support of that, a delay in surgical management might not have a significant effect on prognosis in cases of pediatric patients with blunt intestinal injury. 6

Our focused search did not identify any reports describing successful nonoperative management of mesenteric hematomas in pediatrics with blunt trauma. Some studies concluded that all pediatric blunt trauma patients with isolated mesenteric injuries required an operative management approach, regardless of their hemodynamic status. 1 One study used the argument of mesenteric hematomas that were suspected to have led to adjacent intestinal stenosis, presenting 62 days from the incidence of blunt abdominal trauma. 7

While complications of this nature are unpredictable, concern may be raised for injuries of the mesentery that carry potential for later complications while being hidden to imaging but not to operative exploration. In the case of mesenteric shearing, the technically plausible example would be a traumatic rent formation with risk for internal hernia. However, the presence of only a few case reports presenting scenarios of delayed posttraumatic mesenteric internal hernias speaks for its likely low risk. Nonetheless, it can occur, and less invasive means of exploration are an option. For stable and less severe pediatric blunt abdominal trauma, laparoscopy can offer good, if not better, results, with low risk for missing injuries. 8 9 It can be tempting to conclude that laparoscopy should thus be the management of choice for these patients. Indeed, a tear in the mesentery is not a sight that is seen on CT imaging and is often only inferred from other CT signs and findings. 10 What is important to note, though, is that significant tears will only be expected with more advanced injury scales, injuries that will warrant surgery regardless of whether a true full thickness tear is or is not present. Therefore, CT imaging and the clinical condition of the patient seemingly continue to dictate the need for operative evaluation, including that in the case of laparoscopy for a possible mesenteric rent.

CT with IV contrast remains the imaging of choice for pediatric blunt abdominal trauma with suspicion for intra-abdominal injury. 11 An issue of concern, though, is the radiation involved in the case of pediatric age group patients, and even more so when repeat imaging is performed. The question that naturally follows is whether follow-up imaging could be performed with magnetic resonance imaging or ultrasonography instead. In light of the drawbacks relating to time and cost for magnetic resonance imaging, the candidate modality would be ultrasound. Ultrasound for follow-up of mesenteric hematomas has been reported, though this is limited to case reports. 12 13 However, the follow-up often mentioned is weeks to months after the trauma, after stability is established in the immediate period. Furthermore, it should be noted that evidence for its ability to identify active bleeding is also still rudimentary, with current studies showing promise for efficacy, mainly for contrast-enhanced ultrasonography, but still only in research settings. Based on the data available, perhaps the role for ultrasound in follow-up lies in being an adjunct to clinical monitoring for stable patients, rather than a management-defining factor. Pertinently, patient habitus and location of the mesenteric hematoma must first allow reliable visualization, and technical feasibility is not guaranteed, as was the case with our patient.

To our knowledge, similar detailed reports of successful nonoperative management of isolated mesenteric hematoma are either very few or not present. Nonoperative management can be successful in these patients. However, this claim can only be made for patients who are hemodynamically stable on presentation, without frank peritoneal signs, and remain clinically and hemodynamically stable without deterioration in laboratory or image findings. This is in line with the studies mentioned that advise a more conservative approach to patients with low clinical suspicion for hollow-organ injury. Isolated mesenteric hematoma itself may not routinely require surgery, nor is it necessarily associated with delayed complications that would warrant it. Seeing as this is a case report, it cannot be representative of how this pathology should routinely be approached. Larger, more elaborate studies are required to confidently address these cases.

## Conclusion

In hemodynamically stable patients without considerable peritoneal signs and without findings suggesting deterioration, isolated mesenteric hematoma itself may not routinely require surgery, nor is it necessarily associated with delayed complications that would warrant it.

## Notes

Importance for a Pediatric Surgeon This case highlights that conservative management of isolated mesenteric shear injuries may be safe and effective in hemodynamically stable pediatric patients without peritoneal signs. It emphasizes the need to balance surgical intervention with clinical judgment and evolving evidence, potentially avoiding unnecessary operations.
